# The thrombotic microangiopathies

**DOI:** 10.1007/s00467-007-0616-x

**Published:** 2008-10-01

**Authors:** Lawrence Copelovitch, Bernard S. Kaplan

**Affiliations:** Department of Pediatrics, Division of Nephrology, The Children’s Hospital of Philadelphia, 34th Street and Civic Center Boulevard, Philadelphia, PA 19104 USA

**Keywords:** Thrombotic microangiopathy, Hemolytic uremic syndrome, Thrombotic thrombocytopenic purpura

## Abstract

The term thrombotic microangiopathy (TMA) encompasses a group of conditions that are defined by, or result from, a similar histopathological lesion. Hemolytic uremic syndrome (HUS), thrombotic thrombocytopenic purpura (TTP), and several other conditions are associated with TMA. Distinguishing HUS from TTP is not always possible unless there are specific causes, such as Shiga toxin, *Streptococcus pneumoniae*, or a specific molecular defect such as factor H or ADAMTS13 deficiency. This review describes the forms of HUS/TTP that are not related to Shiga toxin, pneumococcal infection, genetic causes, or ADAMTS13 deficiency. Conditions include HUS/TTP associated with autoimmune disorders, human immunodeficiency virus (HIV) infection, transplantation, malignancy, and medications.

## Introduction

Hemolytic uremic syndrome (HUS) is defined as the triad of microangiopathic hemolytic anemia, thrombocytopenia, and acute renal injury. Thrombotic thrombocytopenic purpura (TTP) is characterized by the pentad of microangiopathic hemolytic anemia, thrombocytopenia, fever, acute renal injury, and neurological abnormalities. Generally, renal manifestations predominate in HUS, and neurological features are important in TTP. It is also clear that there are many types of HUS and TTP that can now be defined not only by these classical criteria but, more precisely, by known etiological factors [1]. It is accepted that there are clinicopathological entities called Shiga toxin HUS, pneumococcal HUS (see the Teaching Article on this entity), and genetic (inherited, familial) HUS with deficiencies of factors H, I, B or membrane cofactor protein. There are also acquired and constitutive deficiencies in the activities of von Willebrand factor (vWF) cleaving protease (ADAMTS13) that result in TTP.

**Fig. 1:**
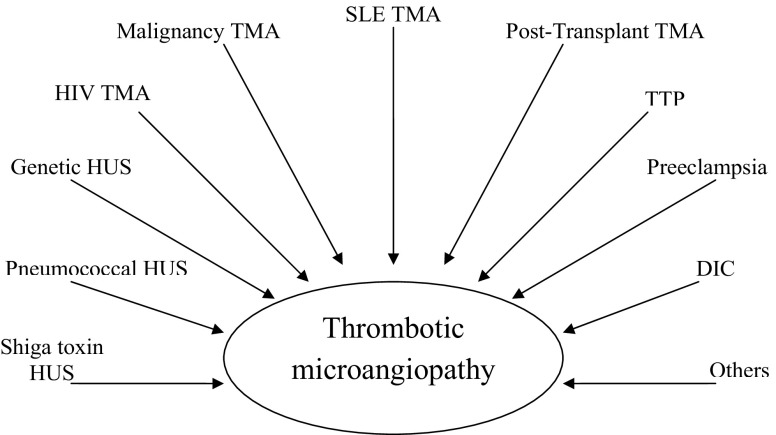
Thrombotic microangiopathies

However, there are other syndromes that do not fall easily under the above rubrics. In 1952 Symmers introduced the all-encompassing term thrombotic microangiopathy (TMA). This term helps us to address some of the ambiguities that defy clinical classification [2]. TMA describes a pattern of arteriolar thrombi, with intimal swelling and fibrinoid necrosis of the vessel wall [3]. TMA in its broadest definition is the histopathological feature of HUS and TTP; however, the composition of the thrombi differs markedly between well-defined types of HUS and TTP. The thrombi in Shiga toxin HUS are rich in fibrin, whereas those in TTP are mainly composed of vWF and degranulated platelets [4]. TMA is also observed in several other conditions, including systemic lupus erythematosus (SLE), malignancy, disseminating intravascular coagulopathy (DIC), and pre-eclampsia (Fig. 1). TMA is a final pathological endpoint that results from a disruption of the normal platelet–endothelial interface [5]. This can occur either through direct vascular endothelial wall damage by Shiga toxin or Thomsen–Friedenreich antigen activation (pneumococcal HUS), or from a defect in normal plasma regulatory systems, such as factor H deficiency or ADAMTS13 deficiency.

Our approach is to classify HUS as: 1. Shiga toxin HUS; 2. Pneumococcal HUS; 3. Genetic HUS; 4. HUS and/or TTP-like forms of TMA not associated with decreased levels of ADAMTS13. In this review we discuss the diverse group of conditions that we refer to as the non-Shiga toxin, non-pneumococcal, non-genetic, non-ADAMTS13-deficient forms of HUS or TTP. To get around this cumbersome designation, we will use the term HUS/TTP to encompass these cases that would be more accurately classified in the broader category of TMA. These rare conditions include HUS/TTP associated with autoimmune disorders, human immunodeficiency virus (HIV) infection, transplantation, malignancy, and medications.

Rigorous classification of TMA is important, because of the implications for treatment. Patients with genetic HUS that result from congenital deficiencies of complement pathway regulators, or congenital TTP that result from a congenital deficiency of ADAMTS13, may benefit from the replacement of these factors through plasma infusions. Patients with acquired TTP may benefit from plasma exchange by the removal of the vWF-cleaving protease inhibitors, thereby restoring ADAMTS13 levels. There is no proven benefit for plasma infusions or plasmapheresis in Shiga toxin HUS or pneumococcal HUS. Furthermore, there is no proven benefit for plasma infusions or plasmapheresis in HUS/TTP associated with autoimmune disorders, human immunodeficiency virus (HIV) infection, transplantation, malignancy, or medications.

## Autoimmune disorders

TMA occasionally occurs in SLE [6–8] and antiphospholipid antibody syndrome (APLS) [9] and rarely in many autoimmune disorders (Table 1).

**Table 1:** Autoimmune disorders associated with TMA

Systemic lupus erythematosus	Antiphospholipid antibody syndrome
Scleroderma	Sjögren syndrome
Mixed connective tissue disease	Dermatomyositis
Rheumatoid arthritis	Ankylosing spondylarthritis
Behçet disease	Polyarteritis nodosa
Myasthenia gravis	Adult Still’s disease
Ulcerative colitis	Idiopathic thrombocytopenic purpura

### Clinical features and diagnosis

Approximately 60 cases of TMA associated with SLE are reported [6–8]. Mirroring the demography of SLE, the majority of the patients are female adolescents or young adults [6]. The onset of SLE often precedes HUS/TTP (>60%); but HUS/TTP may occur simultaneously or precede SLE [6]. From 1–4% of patients with SLE have an episode of HUS/TTP during their illness [7, 10], but one autopsy study found a prevalence of 14%, suggesting that the diagnosis of HUS/TTP may be overlooked [11]. This might occur because HUS/TTP and SLE have overlapping clinical features, including hemolytic anemia, thrombocytopenia, fever, neurological dysfunction, and renal impairment. However, SLE, per se, is not associated with a microangiopathic hemolytic anemia characterized by schistocytes. When hemolytic anemia and thrombocytopenia occur in SLE in the absence of schistocytes, they are usually accompanied by positive findings in a Coombs’ test and by anti-platelet antibodies. A positive result for a Coombs’ test does not exclude the diagnosis of HUS/TTP.

### Pathogenesis

The exact pathogenesis of SLE associated TMA (SLE-TMA) remains unknown and may differ between patients. Zheng et al. [10] reported a severe deficiency of ADAMTS13 in 80% of patients with TTP and in none of those with autoimmune-, transplantation-, malignancy-, medication-, or pregnancy-associated (secondary forms) of TTP. However, only one of the patients with secondary TTP had SLE-TMA. In contrast, in a study of the ADAMTS13 levels of 15 patients with autoimmune disease-associated TMA (SLE, APLS, thyroiditis, psoriasis, Crohn disease), seven had undetectable ADAMTS13 levels, five had normal levels, and three had intermediate levels [12]. Furthermore, seven had inhibitors of the vWF-cleaving protease. In addition, the development of SLE-TMA may be the result of a more generalized autoimmune process that results in direct endothelial injury, with anti-endothelial antibodies [12]. The direct endothelial damage might lead to reduced prostacyclin synthesis, platelet activation and vWF abnormalities, resulting in TMA [8].

APLS autoantibodies are also implicated in the development of SLE-TMA. Although some patients with SLE-TMA have a positive antiphospholipid antibody panel, these antibodies may be found in up to 50% of all patients with SLE [13]. Whether APLS contributes to disease development in some SLE-TMA patients is unclear. Further complicating matters, two patients with primary APLS (without SLE) developed HUS/TTP in association with a severe deficiency of ADAMTS13 and the presence of a vWF-cleaving protease inhibitor [9]. The great difficulty in unraveling the pathogenesis of these conditions is the result of clinically overlapping picture of HUS/TTP, SLE, and APLS, the possibility that these conditions might coexist, and the possibility that ADAMTS13 levels might be depressed in many conditions.

### Treatment

There is no established guideline for the treatment of SLE-TMA. Plasma exchanges, prednisone, and cyclophosphamide are the most frequent choices of therapy [7]. The overall mortality rate among 56 patients with SLE-TMA was 33.9% [6]. In this review the mortality rate of the subset of patients treated with plasmapheresis or plasma exchange was 31.9%, compared with 44.4% in a group not treated with plasmapheresis or plasma exchange. While plasmapheresis may reduce the mortality rate in SLE-TMA, it is not as effective as in TTP, where the mortality rate in treated patients is <10%. Because of the limited number of reported cases of APLS-TMA there is no standard of care [14].

## Human immunodeficiency virus

### Clinical features and diagnosis

HUS/TTP has been reported in several hundred HIV-infected adults [15]. The confounding clinical spectrum of acquired immunodeficiency syndrome (AIDS) with autoimmune thrombocytopenia, myelodysplasia, central nervous system dysfunction, HIV nephropathy, medication exposure, opportunistic infections, and secondary malignancies often makes the diagnosis of HIV-associated TMA (HIV-TMA) confusing [16]. The prevalence of TMA in adult HIV patients is 7% to 35% [17, 18], but it is rare in childhood [19].

### Pathogenesis

There are reports of HIV-TMA with ADAMTS13 deficiency and inhibitors of the vWF-cleaving protease [16], but the ADAMTS13 levels were not measured in most cases. The best evidence for the importance of ADAMTS13 deficiency in HIV-TMA is in the possible response to plasma infusion therapy [20]. Alternatively, the pathogenesis of HIV-TMA may be the result of a primary endothelial injury. Whether the endothelial damage is directly related to viral infection of the microvasculature and renal cells or is indirectly related to altered vasoactive factors, local coagulation defects, inflammatory injury, or direct damage from HIV subunit peptides (Tat, gp120), is unclear [15].

### Treatment

There is no consensus on the optimal treatment of HIV-TMA. Plasmapheresis/plasma exchange is the most widely employed therapy, but the results are mixed [15, 20]. Some clinicians feel that there is no compelling case for any therapy above and beyond routine HIV treatment and supportive care [15].

## Transplantation

### Clinical features and diagnosis

Post-transplantation TMA may be the result of recurrent disease or a de novo event. Recurrent TMA is usually associated with genetic forms of HUS. De novo TMA can occur after hematopoietic stem cell transplantation (HSCT) or solid organ transplantation. The incidence of TMA after allogenic HSCT (HSCT-TMA) is 0.5% to 76% [21]. The estimated prevalence is 8.2%, with a median mortality of 75% [22]. The median onset is 44 days after HSCT (13 to 319 days) [23]. HSCT-TMA is classified into four overlapping subtypes: multifactorial fulminant TMA, conditioning-associated HUS, cyclosporine A-associated nephrotoxicity with microangiopathic hemolytic anemia, and cyclosporine A-associated neurotoxicity with microangiopathic hemolytic anemia [24]. Multifactorial fulminant TMA and cyclosporine A-associated neurotoxicity with microangiopathic hemolytic anemia have poor prognoses, while the courses of condition-associated HUS and cyclosporine A-associated nephrotoxicity with microangiopathic hemolytic anemia are often milder.

Early identification of HSCT-TMA can be difficult. There is clinical overlap with calcineurin inhibitor toxicity, which can also cause red cell fragmentation, thrombocytopenia, renal dysfunction and neurological problems. Furthermore, many of the features of TMA may not be present initially or may be attributed to other causes. In particular, the platelet and red blood cell count may fall because engraftment may not have occurred. Similarly, minor red cell fragmentation is common after bone marrow transplantation, and a developing TMA may be overlooked. Finally, the early renal or neurological manifestations of HUS/TTP may be difficult to discern in ill patients on multiple medications. The incidence of HSCT-TMA may be no greater than in the general population [22], because of the frequent uncertainty of diagnosis, the variability of reported incidences, the potential for mimicry from other transplantation-related complications, and the absence of autopsy findings in most reports.

TMA has been reported after transplantation of all types of solid organs, although the majority of cases were after renal transplantation [25]. The United States Renal Data System has shown an incidence of 0.8% of de novo transplantation-associated TMA in renal transplantation [26], but single-center studies have reported incidences up to 14% [27, 28]. The risk of TMA is highest during the first 3 months after transplantation [26], and 96% of cases occur within 1 year [25]. TMA is usually associated with the use of calcineurin inhibitors [29], but sirolimus [30], vascular rejection [31], and multiple viral agents, including cytomegalovirus infection, have been implicated [32]. Of particular interest, acute vascular rejection can have clinical and histological features similar to those of TMA; therefore, accurate diagnosis is essential, as treatment varies considerably. While TMA is a result of primary, non-antibody mediated, injury to endothelial cells, the specific lesion for vascular rejection appears to be an antibody-mediated endovasculitis. This acute humoral rejection results in the binding of antibodies to donor endothelium, which activate the classical complement pathway, causing deposition of C4d in the peritubular capillaries. C4d immunostaining of the renal biopsy tissue may be informative in cases where the diagnosis is unclear [33].

### Pathogenesis

The risk factors for TMA after HSCT include allogenic transplantation, an unrelated stem-cell donor, acute graft-versus-host disease, calcineurin inhibitors, rapamycin, hepatic veno-occlusive disease, cytomegalovirus, human herpes virus-6, parvovirus B19, and adenovirus [27, 34]. The pathogenesis may relate to underlying endothelial damage associated with radiotherapy, chemotherapy, calcineurin inhibitors, infection and, possibly, graft-versus-host disease. The pathophysiology of TMA after solid organ transplantation is believed to be secondary to an endothelial injury rather than an autoimmune process. ADAMTS13 levels are not markedly depressed in most cases of HSCT-TMA [10, 35] or TMA associated with solid organ transplantation [29]. Regardless, the most important risk factors seem to be calcineurin inhibitors and anti-mTOR agents [27, 30], and the risk is increased when those agents are used together [27].

### Treatment

There is no effective treatment for transplantation-associated TMA. The calcineurin inhibitor may be discontinued, but this may not reverse the TMA and may result in acute graft-versus-host disease [34]. Switching cyclosporine A to tacrolimus, or vice versa, occasionally results in recovery. Plasmapheresis and plasma exchange are currently not considered standards of care [21, 22]. Furthermore, the absence of severe ADAMTS13 deficiency provides no rationale for these potentially harmful interventions.

## Malignancy

### Clinical features and diagnosis

Malignancy associated TMA is mainly seen in adults and is associated with many different adenocarcinomas [34, 36]. Rarely, TMA may be associated with leukemia [37] or lymphoma [38]. Malignancy associated TMA may be manifested at any stage of the disease, from early in the course to widely disseminated cancer. When TMA is the first manifestation of an occult malignancy, patients present with abrupt onset of hemolytic anemia and thrombocytopenia; renal dysfunction is less common than in other forms of HUS/TTP [34].

### Pathogenesis

The pathogenesis of malignancy associated TMA is multifactorial. ADAMTS13 levels in malignancy range from undetectable to normal [10, 36]. Microvascular tumor emboli, tumor procoagulants, monocyte procoagulants, and impaired fibrinolysis may also be implicated [34].

Malignancy associated TMA may be triggered by chemotherapeutic agents (mitomycin C and gemcitabine). However, the occurrence of HUS/TTP in the absence of these agents suggests that some cases result from a paraneoplastic phenomenon [37–39]. Radiation exposure and opportunistic infections such as cytomegalovirus infection [40] contribute to the difficulty of assigning a cause. Furthermore, in disseminating malignancies, a syndrome resembling HUS/TTP can be mimicked by DIC.

### Treatment

Treatment of the underlying cancer is the mainstay of therapy. When a chemotherapeutic agent is a suspected cause, attempts to decrease or discontinue the drug are considered. There is no role for plasmapheresis.

## Medications

Many commonly prescribed medications, vaccines, illicit drugs, and exogenous substances are reported to be associated with TMA [41]. Most of the case reports are difficult to evaluate because of the possibility of a chance association, concomitant disease states, and exposure to multiple medications [41, 42]. The five most commonly reported TMA-associated agents are cyclosporine A, tacrolimus, mitomycin C, quinine, and ticlopidine.

The calcineurin inhibitors are associated with TMA after solid organ or hematopoietic stem-cell transplantation. They also cause TMA in Behçet disease [43] and systemic sclerosis [44]. Healthy rhesus monkeys exposed to tacrolimus developed anemia with schistocytes, thrombocytopenia, and renal microangiopathy [45].

The incidence of transplant associated-TMA ascribed to cyclosporine A is 13% [28], and to tacrolimus it is 1% to 4.7% [46]. The onset of TMA may be associated with supra-therapeutic or therapeutic levels of cyclosporine A [41]. Cyclosporine A-associated TMA is often confined to the kidney but may be associated with hematological derangements. Endothelial toxicity induced directly by calcineurin inhibitors is mediated by thromboxane-induced vasoconstriction, alterations in prostacyclin synthesis, increased renin activity, increased endothelin secretion, and the reduced formation of activated protein C [42, 47] but not ADAMTS13 deficiency [48]. Treatment is withdrawal of the offending agent, although not all patients respond.

Many antineoplastic agents (bleomycin, cisplatin, gemcitabine) are associated with TMA, with mitomycin C most frequently reported [41, 42]. It is likely that mitomycin C causes direct endothelial damage [49]. Treatment with plasma exchange is not effective, and overall prognosis is extremely poor [42].

Quinine, as a medication or food additive, is associated with TMA. In one series, 57% of cases of drug-associated HUS/TTP were ascribed to quinine. Patients exposed to quinine may develop autoantibodies against platelets, granulocytes, lymphocytes, and endothelial cells [42] but not against ADAMTS13 [35]. Quinine-associated TMA is not dose related, and re-exposure to one dose after many years can result in recurrence [42]. Treatment is withdrawal of the quinine. Use of plasmapheresis is based on uncontrolled trials.

The incidence of ticlopidine (an anti-platelet agent) associated TMA is 0.02–0.06% [50, 51]. In one series most patients had depressed ADAMTS13 activity and vWF-cleaving protease inhibitors [52]. Furthermore, all the patients completely recovered after discontinuation of ticlopidine and treatment with plasmapheresis. In contrast, a separate series of patients with ticlopidine-associated HUS/TTP had normal to near normal ADAMTS13 levels [53]. In addition, ticlopidine was shown to induce apoptosis directly in cultured human microvascular endothelial cells by disrupting the normal endothelial cell–extracellular matrix interactions. These observations suggest that there are several causal pathways in ticlopidine-associated TMA. Clopidogrel, a related compound, has replaced ticlopidine because of fewer side-effects. Clopidogrel less commonly causes HUS/TTP [42]. Treatment of ticlopidine-associated HUS/TTP involves cessation of the medication, and plasma exchange [42, 51].

## Unclassified

Many conditions are listed as possible causes of HUS: infectious mononucleosis, *Coxiella burnetii*, group A beta hemolytic streptococcus, *Salmonella typhi*, hepatitis A, Kawasaki disease, and many others. Pregnancy has also been proposed as a risk factor, but this is complicated by pre-eclampsia, hemolytic anemia, elevated liver enzymes and low platelets (HELLP) syndrome, idiopathic TTP, factor H deficiency, and *E. coli* 0157:H7 infection during or after pregnancy.

## Conclusion

Considerable progress has been made in the understanding of HUS, TTP, and TMA. The recognition of the importance of the Shiga toxins, neuraminidase activity, abnormalities in the vWF-cleaving protease, and regulatory elements of the complement cascade, has elevated some of these conditions from syndrome to the status of clinico-pathological diseases. The pathophysiology of many of the secondary causes of TMA remains unknown. Whether the ADAMTS13 activity will truly distinguish HUS from TTP in many problematic cases remains to be seen.

## Questions: (Answers appear after the reference list)

1. Which of the following should *not* be routinely treated by plasma exchange or infusion?
  - TTP
  - Factor H deficiency-associated HUS
  - Ticlopidine-associated HUS
  - TMA after hematopoietic stem cell transplantation
  - Congenital TTP (Upshaw–Schulman syndrome)
2. Which of the following contributes to the difficulty in establishing the diagnosis of TMA after hematopoietic stem cell transplant?
  - Clinical overlap with calcineurin toxicity
  - Delayed bone marrow engraftment
  - The frequency of minor red cell fragmentation
  - Neurological and renal dysfunction can occur for many other reasons
  - All of the above
3. Which of the following is true regarding malignancy associated TMA.
  - Is a leading cause of HUS in children
  - Usually associated with lymphoma or leukemia
  - Can always be attributed to a chemotherapeutic agent
  - Renal dysfunction is less common than in other forms of secondary HUS/TTP
  - TMA never precedes the diagnosis of malignancy
4. Which of the following is true regarding SLE-associated HUS/TTP?
  - The onset of SLE usually precede HUS/TTP
  - Is always associated with APLS
  - A positive finding in a Coombs’ test excludes the diagnosis of HUS/TTP
  - Has a mortality rate of <10% when treated with plasmapheresis
  - Is always associated with a severe deficiency of ADAMTS13
5. Which of the following is *not* true regarding TMA after solid-organ transplantation?
  - Is most frequently associated with renal transplantation
  - Is usually diagnosed more than 12 months after transplantation
  - Is often confused with vascular rejection
  - Is usually associated with calcineurin inhibitors
  - ADAMTS13 levels are frequently normal
